# Proof of Concept and Validation of Single-Camera AI-Assisted Live Thumb Motion Capture

**DOI:** 10.3390/s25154633

**Published:** 2025-07-26

**Authors:** Huy G. Dinh, Joanne Y. Zhou, Adam Benmira, Deborah E. Kenney, Amy L. Ladd

**Affiliations:** 1 Department of Orthopaedic Surgery, Stanford University, Stanford, CA 94305, USA; hdinh98@stanford.edu (H.G.D.); joanne.zhou2@emory.edu (J.Y.Z.); abenmira@stanford.edu (A.B.); dkenney@stanford.edu (D.E.K.); 2 Department of Orthopaedics, Emory University, Atlanta, GA 30322, USA

**Keywords:** artificial intelligence, motion capture, motion analysis, hand measurement, thumb measurement, range of motion, hand pose estimation, telemedicine, carpometacarpal joint

## Abstract

Motion analysis can be useful for multiplanar analysis of hand kinematics. The carpometacarpal (CMC) joint has been traditionally difficult to capture with surface-based motion analysis but is the most commonly arthritic joint of the hand and is of particular clinical interest. Traditional 3D motion capture of the CMC joint using multiple cameras and reflective markers and manual goniometer measurement has been challenging to integrate into clinical workflow. We therefore propose a markerless single-camera artificial intelligence (AI)-assisted motion capture method to provide real-time estimation of clinically relevant parameters. Our study enrolled five healthy subjects, two male and three female. Fourteen clinical parameters were extracted from thumb interphalangeal (IP), metacarpal phalangeal (MP), and CMC joint motions using manual goniometry and live motion capture with the Google AI MediaPipe Hands landmarker model. Motion capture measurements were assessed for accuracy, precision, and correlation with manual goniometry. Motion capture demonstrated sufficient accuracy in 11 and precision in all 14 parameters, with mean error of −2.13 ± 2.81° (95% confidence interval [CI]: −5.31, 1.05). Strong agreement was observed between both modalities across all subjects, with a combined Pearson correlation coefficient of 0.97 (*p* < 0.001) and an intraclass correlation coefficient of 0.97 (*p* < 0.001). The results suggest AI-assisted live motion capture can be an accurate and practical thumb assessment tool, particularly in virtual patient encounters, for enhanced range of motion (ROM) analysis.

## 1. Introduction

**Figure 1:**
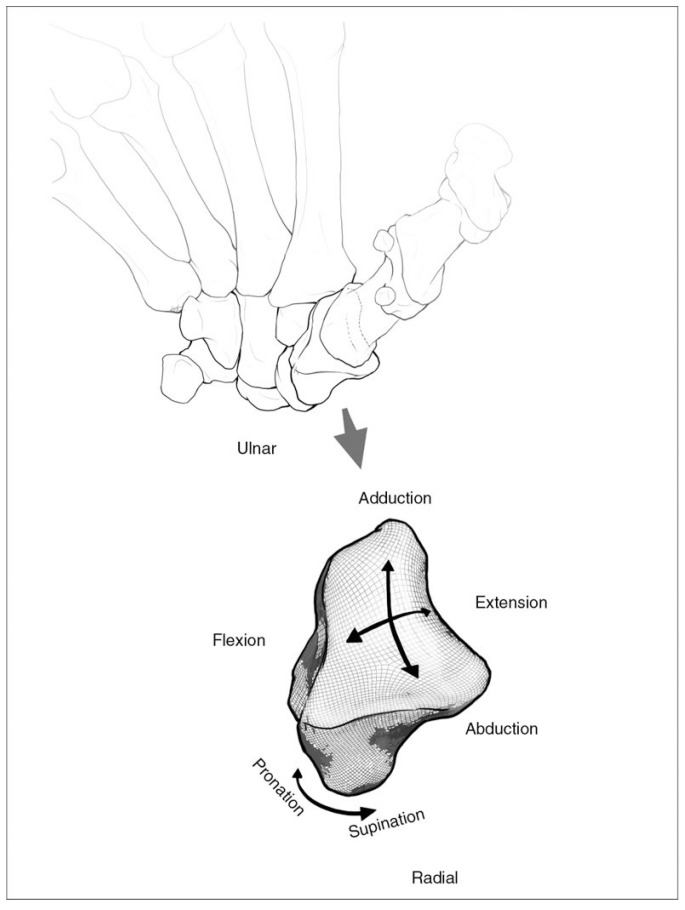
Bony anatomy and motion arcs of the carpometacarpal (CMC) joint. Published with kind permission of © S. Hegmann 2014 [9]. All rights reserved.

The unique anatomy of the carpometacarpal (CMC) joint imparts on humans the ability to perform specialized tasks that have become crucial to daily living. The biconcave saddle osseous anatomy of the thumb CMC joint allows for both dexterity and stability, allowing for pinch, grasp, and release required for activities of daily life (Figure 1). Disease of the CMC joint can thus be very debilitating, with thumb CMC osteoarthritis (OA) being the second most common degenerative disease of the hand [1,2]. Earlier stages of CMC OA can be managed non-operatively but may eventually require surgical intervention as symptom severity increases with disease progression [3,4]. Among the surgical options available, various thumb CMC arthroplasty techniques exist to treat CMC OA, with a focus on maintaining motion, preventing thumb shortening, improving pinch strength, and limiting postoperative recovery time [5,6,7]. However, at present, none are universally considered superior to another, and many studies have found that full strength may not be fully restored [8,9,10].

Increasingly in modern life, texting and typing have become commonplace daily tasks that require significant loads across the thumb [11]. Approximately 307 million people in the United States, or 92% of the population, own smartphones and use them at an average reported screen time of 4 h and 25 min per day [11]. Previous studies have found that texting places the thumb at increased loads, and the incidence of peak force is linked to high flexion angle of the interphalangeal (IP) joint and to thumb opposition of the metacarpal phalangeal (MP) joint [11]. To adequately evaluate and provide rehabilitation for thumb CMC arthroplasty, it is imperative to have a complete understanding of thumb motion, particularly ones that are involved in these common modern tasks.

The non-phasic, three-dimensional nature of thumb function, which involves fine manipulation for targeted activity, has historically been difficult to model and quantify [12]. Thumb motion is typically evaluated in clinical practice using manual goniometry, however it requires special training for accurate and consistent measurements and lacks the ability to record fluid motion [13,14]. While smartphone goniometry has more recently been developed to simplify measurements, goniometry inherently presents challenges for studies seeking to evaluate multiplanar movement of complex joints, particularly in the carpus [15].

Motion capture has previously been explored for potential applications in thumb motion [16,17,18]. While 3D motion capture with reflective markers has traditionally been the gold standard for biomechanical evaluation of movements, this approach poses several logistical hurdles in the clinical workflow. Often requiring the setup of multiple cameras, physical markers, a designated facility, and technical resources to perform post processing and data analysis, these barriers make impractical in a clinical setting [19,20]. Scalability therefore remains a pertinent challenge with current motion capture modalities.

To address these challenges, we propose a single-camera, AI-assisted hand landmarker task platform capable of providing real-time range of motion (ROM) data during live recordings. The proposed setup does not require special equipment and facilities, nor is it confined by need for lab-based analysis or trained professionals for accurate measurement of motion. We therefore aim to evaluate its performance against manual goniometry to assess its viability as an alternate or supplemental tool for virtual clinic applications in disease management and physical rehabilitation.

## 2. Materials and Methods

We performed our initial pilot study on healthy volunteers with the following inclusion criteria: individuals with no prior injury or surgery to the affected limb. Participants were given the option to choose either hand for this study provided it satisfies the inclusion criteria. All participants consented prior to data collection.

For the purposes of this study, manual goniometry was considered the gold standard for thumb posture measurements. Goniometry measurements of the thumb were based on the hand impairment evaluation guidelines set by the American Society of Hand Therapists (ASHT) [21]. The following active isolated thumb movements evaluated were as follows: flexion/extension of the interphalangeal (IP) joint, flexion/extension of the metacarpal phalangeal (MP) joint, radial abduction/adduction of the thumb at the carpometacarpal (CMC) joint, and thumb palmar abduction/adduction at the CMC joint. Each task was performed and measured three times.

**Figure 2:**
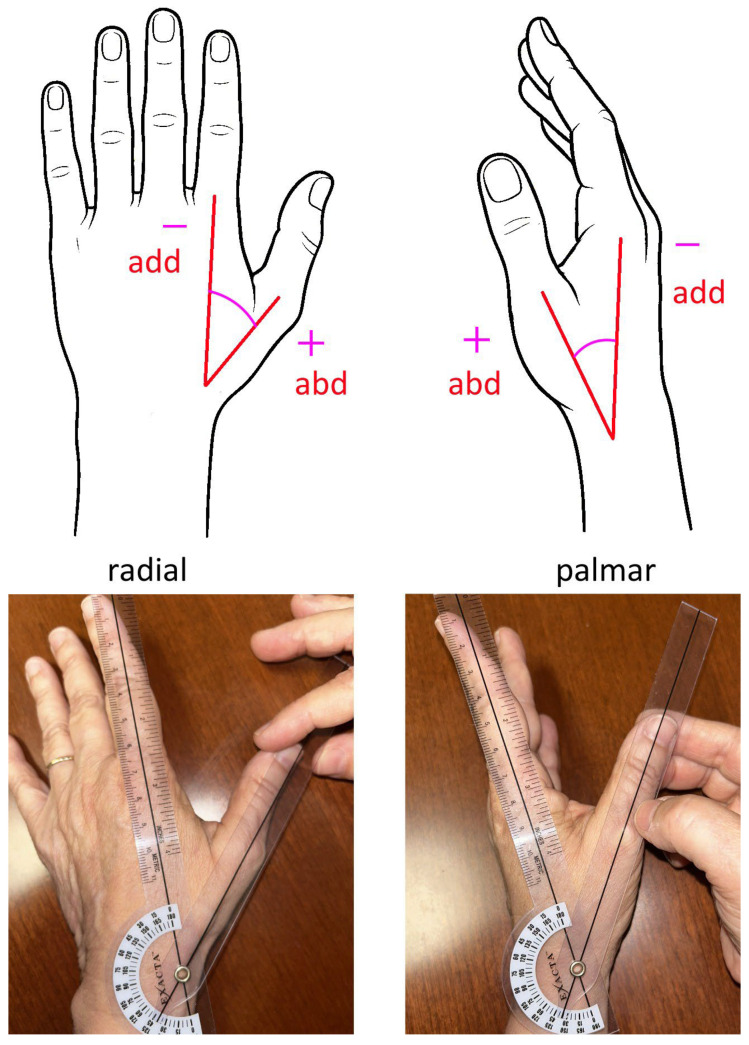
Method for measuring CMC abduction/adduction angle by manual goniometry. The lines forming the angle are the first and second metacarpal. The angle can be further characterized as in the radial or palmar direction.

For IP and MP joint motions, zero degrees was defined as full extension with no hyperextension. CMC motion was defined as movement of the first metacarpal relative to the second metacarpal (Figure 2).

**Figure 3:**
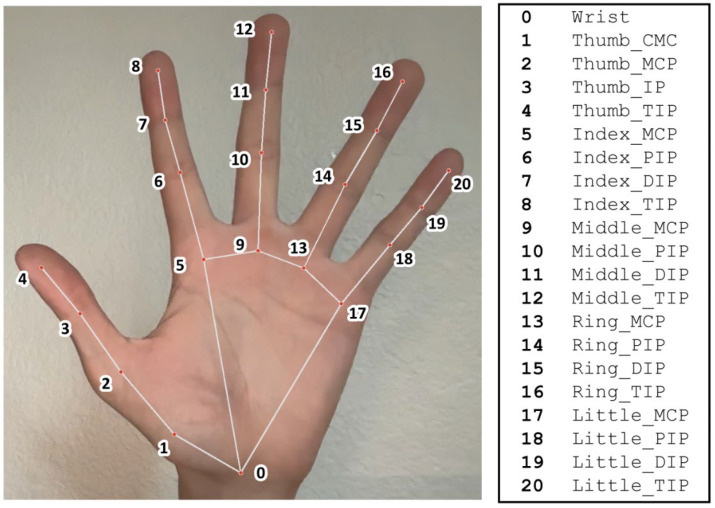
A diagram showing the 21 MediaPipe hand landmarks.

Automatic calculation of movements was performed based on AI-assisted landmark tracking using the Google AIMediaPipe Hand Landmarker model (Mountain View, CA, USA) at the latest version as of May 2025. We executed the model in livestream mode using Python 3.12 to obtain 3D coordinates of the 21 landmarks in the solution (Figure 3). These landmarks were used to calculate target angles during the 4 isolated thumb movements throughout their complete range of motion.

For automatic calculation of IP and MP joint angles, we use the following method with the available hand landmarks:

(1) $$\theta =\cos^{-1}\begin{array}{c} \left(\frac{\left(a-b\right)\cdot \left(c-b\right)}{\left|a-b\right|\cdot \left|b-c\right|}\right) \end{array}$$

(2) $$\;\left|a-b\right|=\sqrt{{{\left|a-b\right|}_{x}}^{2}+{{\left|a-b\right|}_{y}}^{2}+{{\left|a-b\right|}_{z}}^{2}}\;$$

where *θ* is the angle formed by hand landmarks *a*, *b*, *c* at landmark *b*.

We assign landmarks a=1, b=2, c=3 for MP and a=2, b=3, c=4 for IP joint angle calculations, respectively. For automatic calculation of CMC joint angles, we use the following method:

(3) $$\begin{array}{c} if\;v=\left[a_{x}-b_{x},\;a_{y}-b_{y},\;a_{z}-b_{z}\right] \end{array}$$

(4) $$\mathrm{and}\;u=\left[c_{x}-d_{x},\;c_{y}-d_{y},\;c_{z}-d_{z}\right]\;$$

(5) $$\begin{array}{c} then\;\theta =\cos^{-1}\left(\frac{\left(v-u\right)\cdot \left(v-u\right)}{\left|v-u\right|\cdot \left|v-u\right|}\right) \end{array}$$

(6) $$\left|v-u\right|=\sqrt{{{\left|v-u\right|}_{x}}^{2}+{{\left|v-u\right|}_{y}}^{2}+{{\left|v-u\right|}_{z}}^{2}}$$

where *θ* is the angle formed by extended lines of hand landmarks *a*, *b* and *c*, *d*.

We assign landmarks a=1, b=2, c=0, d=9 for all CMC joint angle calculations.

Zero positions for both were defined under the same methodology used for manual goniometry. From these calculations, we subsequently extracted a total of 14 measurement parameters based on the mean and standard deviation (mean ± SD) of the isolated movements (Table 1).

In the first component of the experimental procedure, participants were asked to perform each of the 4 isolated thumb motions: IP flexion/extension, MP flexion/extension, CMC radial abduction/adduction, and CMC palmar abduction/adduction. IP and MP movements began at full extension without hyperextension and ended at complete flexion. CMC movements began at full adduction and ended at full abduction. Trained professional examiners took measurements at the beginning and end of each motion. Participants performed each motion three times to their maximum ranges to yield a mean ± SD describing the start and end of their ROMs.

In the second component, participants were instructed to sit in front of a laptop with a built-in web camera (Apple Macbook Air, 13-inch, M3) that mimicked an online clinic visit. Participants received live instructions throughout the encounter to help maintain appropriate positioning. The 4 isolated thumb motions were performed to yield a total of 6 recordings per participant:

(1) Thumb IP flexion/extension × 3, palm facing the camera;
(2) Thumb MP flexion/extension × 3, palm facing the camera;
(3) Thumb CMC radial abduction/adduction × 3, palm facing the camera;
(4) Thumb CMC radial abduction/adduction × 3, dorsum facing the camera;
(5) Thumb CMC palmar abduction/adduction × 3, palm facing the camera;
(6) Thumb CMC palmar abduction/adduction × 3, dorsum facing the camera.

**Figure 4:**
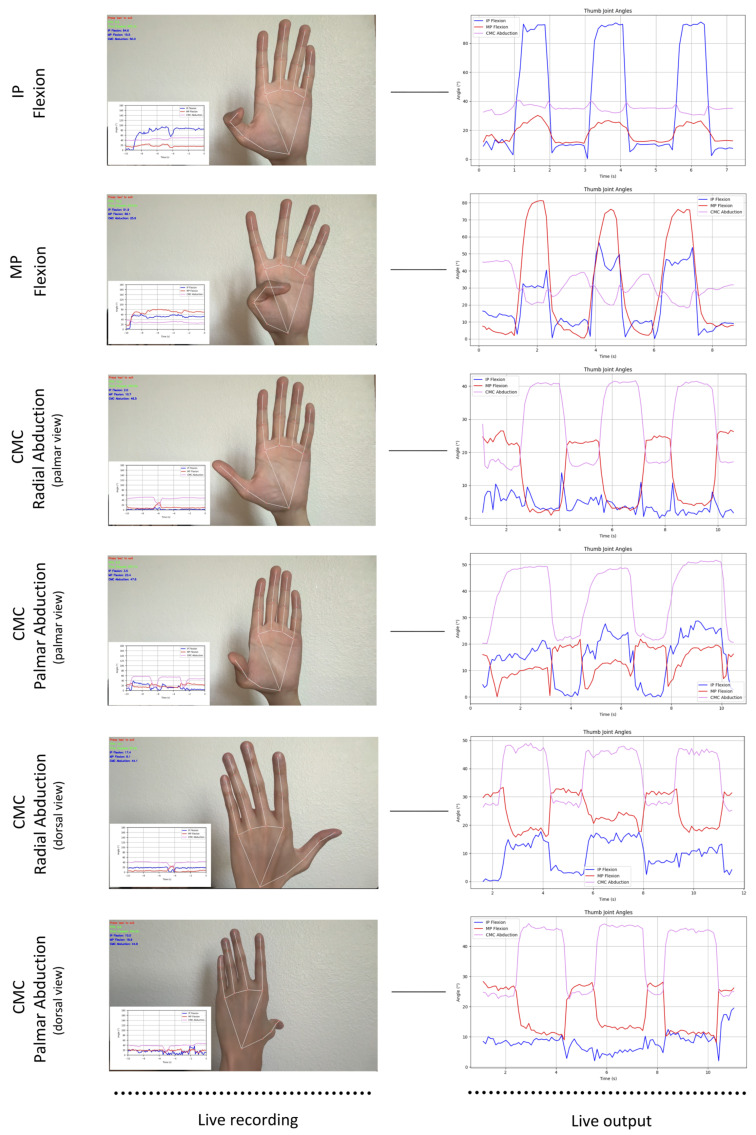
The 4 isolated thumb motions with live recording display and graphical output performed over 6 recordings. Time elapsed from measurement is represented on the x-axis, and the angle calculation is represented on the y-axis. The blue line quantifies IP flexion, the red line quantifies MP flexion, and the violet line quantifies CMC abduction.

Live on-screen angle measurements on screen were extracted at 30 frames per second and visualized in graphical form (Figure 4). Mean ± SD data were subsequently obtained at the start and end of the range of motions (ROM).

Measurement data between manual goniometry and live motion capture were compared to validate motion capture for calculation of the extracted parameters. The accuracy of live motion capture was assessed by calculating the mean error against goniometry for the 14 extracted parameters. Both accuracy and reliability were assessed by determining the 95% confidence interval (CI) of the mean error. We defined an accurate measurement as a mean error of zero contained within the 95% CI. Appropriately precise measurement was defined as a 95% CI smaller than 10°. Distribution of deviations were visualized with Bland–Altman analysis. Pearson’s correlation coefficient (PCC) and intraclass correlation coefficient (ICC) were calculated to examine reliability of measurements within and across subjects. Statistical analysis was performed using Python 3.12.

## 3. Results

### 3.1. Participants

We measured two left and three right hands of five participants. The participant group consisted of three females and two males, all of whom were right-handed with no prior injury or surgery to the included extremity. Subject age ranged from 22 to 63, with a mean age of 33.2 ± 17.0. All participants completed the experimental protocol without complications, and live kinematic data was successfully extracted from all livestream recordings.

### 3.2. Motion Capture Results

**Figure 5:**
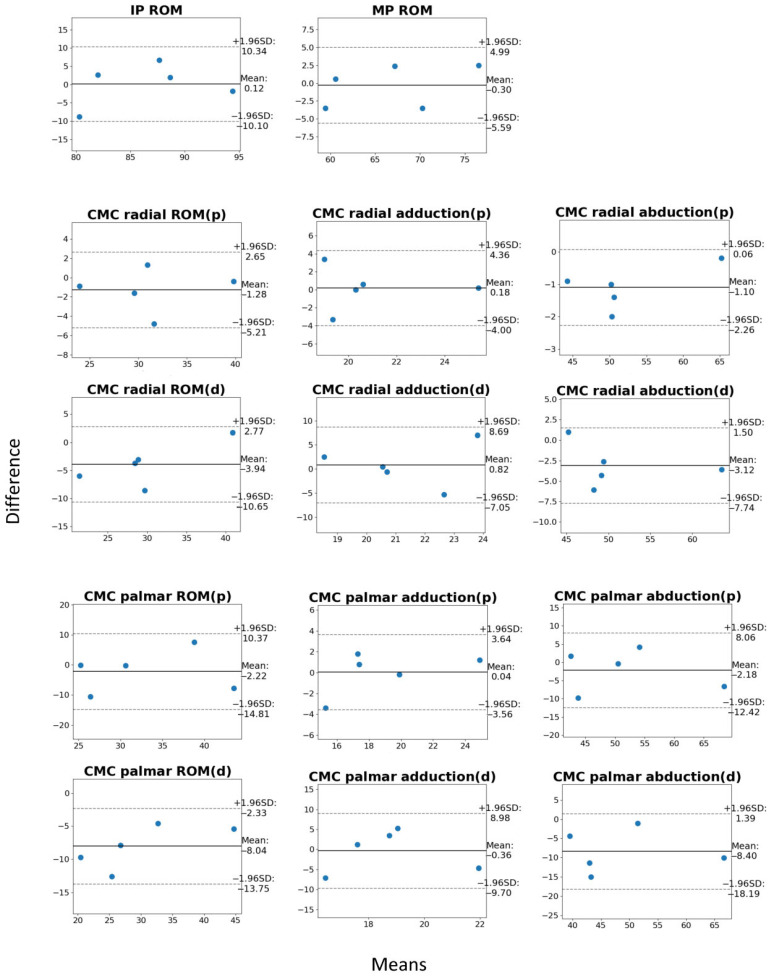
Bland–Altman plots of the measured parameters. Each dot represents a single subject’s calculated mean. Mean between manual goniometry and live motion capture is represented on the x-axis, and motion capture difference from the mean is represented on the y-axis. Mean difference is represented as the bold black line, and limits of agreement are represented with dotted lines.

Calculated mean errors between manual goniometry and live motion capture are represented in numerical form in Table 2 and in Bland–Altman plots in Figure 5. The mean error of all 14 parameters was −2.13 ± 2.81° (95% CI: −5.31, 1.05). A mean error of zero was encompassed by 11 of the 14 parameter 95% CI’s, demonstrating acceptable accuracy. Measurements of IP flexion/extension, MP flexion/extension, CMC radial adduction, and CMC palmar adduction performed exceptionally well, achieving mean errors of less than ±1°. Measurements of CMC radial abduction (palmar view) and overall CMC radial range of motion also performed particularly well, achieving mean errors of less than ±2°. Greatest mean errors were found in CMC palmar abduction (dorsal view) and CMC palmar range of motion (dorsal view) measurements, with values at −8.40 ± 2.81° and −8.04 ± 4.12°, respectively. All 14 parameters furthermore produced 95% CI’s that were smaller than 10°, demonstrating acceptable precision. The best performance by this metric was observed in measurements of CMC radial abduction in both palmar and dorsal views, which achieved 95% CI’s spanning 4.12° and 3.98°, respectively. The largest 95% CI, in contrast, was found in the CMC palmar range of motion (dorsal view) measurement, which spanned 9.32°.

Excellent agreement was observed overall between measurement modalities as shown in Table 3. A combined PCC of 0.974 and ICC of 0.974 were achieved across all subjects, with individual subject PCCs found to be no lower than 0.96 and ICCs no lower than 0.953.

## 4. Discussion

Our proof-of-concept study demonstrates that a single camera-based AI-assisted motion capture device can deliver near-real-time thumb kinematics with accuracy (mean error = −2.13 ± 2.81°), precision (95% CI: −5.31, 1.05), and reliability (ICC = 0.97) that are on par with manual goniometry. The findings further current literature on validating motion capture systems for the hand and provide insights on their potential applications in virtual hand clinics.

Previous studies of pose estimation frameworks have primarily focused on finger phalangeal or composite finger motion using prerecorded videos or still images. In healthy volunteers, hand-tracking pipelines based on OpenPose reported absolute errors of ≤11° for 2D finger joint angles [22]. A still image protocol employing a similar MediaPipe-based framework used in our present study achieved clinically acceptable agreement for 75% of hand parameters [23]. Our study not only substantiates these findings but also extends their validity to a livestream approach to capture base of thumb motion which has traditionally been very challenging to quantify.

A key differentiator of our study is the use of the MediaPipe landmarker task platform in livestream mode with instantaneous calculation and on-screen feedback. Prior still image approaches require pausing of motions at the end ranges, capturing of photographs, and subsequent offline processing. This can result in workflow latency and potential sampling bias toward extreme positions [24]. Real-time feedback, in contrast, allows the examiner to guide the patient dynamically, detect tracking failures immediately, and acquire continuous signals across the entire motion arc, more accurately simulating natural motion. This feature is especially pertinent for identifying pathological motion arcs during functional tasks such as texting or pinching.

Our livestream motion tracking methodology addresses the current limitations of both remote physical examination and traditional 3D motion capture. Previous attempts at remote goniometry using smartphone photography, while convenient in the telemedicine setting, have been susceptible to drops in reliability when patients position the camera themselves without continuous directed guidance [24]. Traditional 3D motion capture, on the other hand, carries the logistical burden of laboratory-grade equipment, facilities, and software that limits feasibility for widespread clinical use. Our setup requires no peripheral sensors, markers, or calibration objects, which reduce costs, simplifies data acquisition. Furthermore, the setup does not require special facilities to deploy. These combined features make it a more practical approach that can seamlessly integrate into the clinical workflow. The ability to readily measure function thumb motion is critical to rehabilitation after surgery or injury, inform the design of implant arthroplasty, and may afford the development of objective outcome indices after surgical treatments.

An unexpected finding was the systematic underestimation of palmar abduction when the thumb was recorded in the dorsal view (mean error = −8.40 ± 2.81°). Visual inspection of the recordings suggests that occlusion of the thenar eminence by the second metacarpal and limited depth cues hinder the model’s ability to resolve the CMC plane when viewed dorsally. Conversely, radial abduction measured dorsally exhibited both the smallest confidence interval (±3.98°) and negligible bias. These results corroborate recent simulation work indicating that optimal camera view is joint and movement specific [25].

We considered measuring palmar abduction from the ulnar or radial side to minimize the need for depth perception. However, preliminary testing interestingly found a palmar view yielded similar measurements to manual goniometry such that there was minimal need view this motion in parallel to the screen, in contrast to radial abduction. As the model training set likely consisted of disproportionately more frames from the palmar view, we were thus able to achieve sufficient adjustment for depth based on the camera settings.

The protocol used in our present study contains several limitations. Our sample comprised only five asymptomatic adults and has not been validated on altered anatomy typical of CMC OA and other hand diseases. The studied parameters were additionally limited to standardized self-paced motions measured at the maximum end ranges and did not include other clinically relevant metrics such as angular velocity and coupled motion patterns motions common in functional tasks. Dynamic analysis that includes data points between the end ranges is accordingly a necessary validation step prior to widespread adoption of the AI-assisted motion capture tool evaluated in our present study. Future directions should therefore focus on fine tuning with additional layers on top of the existing model to further reduce error, with targeted validation of full motion arcs against traditional 3D motion capture to quantify the previously mentioned metrics.

## 5. Conclusions

Many logistical obstacles have prevented wider adoption of motion capture as a physical examination tool. This study establishes that a single-camera, markerless AI-assisted motion capture is feasible for clinical and research applications involving the thumb. The model demonstrated excellent agreement with manual goniometry and contains a viable pre-existing framework for future thumb pose estimation studies. Its potential is ultimately most promising in the virtual clinic space as not only convenient but also reliable and versatile.

## Figures and Tables

**Table 1:** Description of the 14 clinical measurement parameters.

Parameter Name	Parameter Description	Live Motion Capture Measurement	Manual Goniometry Measurement
IP	IP ROM, palmar view	Angle between 2, 3, 4	Angle between dorsal midline of distal and proximal phalanx
MP	MP ROM, palmar view	Angle between 1, 2, 3	Angle between dorsal midline of proximal phalanx and 1st metacarpal
CMC_r_add_p	CMC radial adduction, palmar view	Subtended angle between extended vectors 1, 2 and 0, 9	Angle between dorsal midline of 1st and 2nd metacarpal
CMC_r_add_d	CMC radial adduction, dorsal view	Subtended angle between extended vectors 1, 2 and 0, 9	Angle between dorsal midline of 1st and 2nd metacarpal
CMC_r_abd_p	CMC radial abduction, palmar view	Subtended angle between extended vectors 1, 2 and 0, 9	Angle between dorsal midline of 1st and 2nd metacarpal
CMC_r_abd_d	CMC radial adduction, dorsal view	Subtended angle between extended vectors 1, 2 and 0, 9	Angle between dorsal midline of 1st and 2nd metacarpal
CMC_r_p	CMC radial ROM, palmar view	Subtended angle between extended vectors 1, 2 and 0, 9	Angle between dorsal midline of 1st and 2nd metacarpal
CMC_r_d	CMC radial ROM, dorsal view	Subtended angle between extended vectors 1, 2 and 0, 9	Angle between dorsal midline of 1st and 2nd metacarpal
CMC_p_add_p	CMC palmar adduction, palmar view	Subtended angle between extended vectors 1, 2 and 0, 9	Angle between dorsal midline of 1st and 2nd metacarpal
CMC_p_add_d	CMC palmar adduction, dorsal view	Subtended angle between extended vectors 1, 2 and 0, 9	Angle between dorsal midline of 1st and 2nd metacarpal
CMC_p_abd_p	CMC palmar abduction, palmar view	Subtended angle between extended vectors 1, 2 and 0, 9	Angle between dorsal midline of 1st and 2nd metacarpal
CMC_p_abd_d	CMC palmar abduction, dorsal view	Subtended angle between extended vectors 1, 2 and 0, 9	Angle between dorsal midline of 1st and 2nd metacarpal
CMC_p_p	CMC palmar ROM, palmar view	Subtended angle between extended vectors 1, 2 and 0, 9	Angle between dorsal midline of 1st and 2nd metacarpal
CMC_p_d	CMC palmar ROM, dorsal view	Subtended angle between extended vectors 1, 2 and 0, 9	Angle between dorsal midline of 1st and 2nd metacarpal

**Table 2:** Comparison of measured parameters between manual goniometry and live motion capture.

Parameter Name	Motion Capture (mean ± SD)	Manual Goniometry (mean ± SD)	Mean Error (mean ± SD)	95% CI of Mean Error
IP	86.7 ± 3.5	86.5 ± 0.9	0.12 ± 3.70	(−4.07, 4.31)
MP	66.7 ± 2.3	67.0 ± 1.3	−0.30 ± 3.18	(−3.91, 3.31)
CMC_r_add_p	21.0 ± 2.0	20.8 ± 1.4 *	0.18 ± 2.73	(−2.91, 3.28)
CMC_r_add_d	21.7 ± 1.3	20.8 ± 1.4	0.82 ± 2.16	(−1.63, 3.27)
CMC_r_abd_p	51.6 ± 1.4	52.7 ± 1.2 *	−1.10 ± 1.82	(−3.16, 0.96)
CMC_r_abd_d	49.5 ± 1.1	52.7 ± 1.2	−3.12 ± 1.73	(−5.08, −1.16)
CMC_r_p	30.5 ± 2.6	31.8 ± 1.9 *	−1.28 ± 3.35	(−5.06, 2.51)
CMC_r_d	27.9 ± 1.8	31.8 ± 1.9	−3.94 ± 2.80	(0.76, 7.11)
CMC_p_add_p	19.0 ± 2.3	18.9 ± 1.1 *	0.04 ± 2.72	(−3.04, 3.12)
CMC_p_add_d	18.6 ± 2.4	18.9 ± 1.1	−0.36 ± 2.69	(−3.40, 2.68)
CMC_p_abd_p	50.8 ± 1.1	53.0 ± 1.6 *	−2.18 ± 2.02	(−4.47, 0.11)
CMC_p_abd_d	44.6 ± 2.0	53.0 ± 1.6	−8.40 ± 2.81	(−11.58, −5.22)
CMC_p_p	31.8 ± 2.8	34.0 ± 2.0 *	−2.22 ± 3.52	(−6.20, 1.76)
CMC_p_d	26.0 ± 3.5	34.0 ± 2.0	−8.04 ± 4.12	(−12.70, −3.38)

* All manual goniometer measurements involving CMC motion were performed from the dorsal view as per clinical guidelines.

**Table 3:** Correlation between manual goniometry and live motion capture measurements. All values were significant at *p* < 0.001.

Subject	Intraclass Correlation Coefficient	Pearson Correlation Coefficient
1	0.966	0.977
2	0.958	0.977
3	0.970	0.989
4	0.962	0.973
5	0.953	0.967
Total	0.974	0.974

## Data Availability

The datasets generated and/or analyzed during this ongoing study are not currently available per the experimental protocol approved by the Institutional Review Board.

## Abbreviations

The following abbreviations are used in this manuscript:

IP	Interphalangeal
MP	Metacarpal phalangeal
CMC	Carpometacarpal
ROM	Range of motion
ICC	Intraclass correlation coefficient
PCC	Pearson correlation coefficient
CI	Confidence interval
OA	Osteoarthritis

## References

[1] Athlani L., De Almeida Y.-K., Martins A., Seaourt A.-C., Dap F. (2023). Thumb Basal Joint Arthritis in 2023. Orthop. Traumatol. Surg. Res..

[2] Bakri K., Moran S.L. (2015). Thumb Carpometacarpal Arthritis. Plast. Reconstr. Surg..

[3] Zhang W., Doherty M., Leeb B.F., Alekseeva L., Arden N.K., Bijlsma J.W., Dinçer F., Dziedzic K., Häuselmann H.J., Herrero-Beaumont G. (2007). EULAR Evidence Based Recommendations for the Management of Hand Osteoarthritis: Report of a Task Force of the EULAR Standing Committee for International Clinical Studies Including Therapeutics (ESCISIT). Ann. Rheum. Dis..

[4] Kloppenburg M., Kroon F.P., Blanco F.J., Doherty M., Dziedzic K.S., Greibrokk E., Haugen I.K., Herrero-Beaumont G., Jonsson H., Kjeken I. (2019). 2018 Update of the EULAR Recommendations for the Management of Hand Osteoarthritis. Ann. Rheum. Dis..

[5] Pomares G., Delgrande D., Dap F., Dautel G. (2016). Minimum 10-Year Clinical and Radiological Follow-up of Trapeziectomy with Interposition or Suspensionplasty for Basal Thumb Arthritis. Orthop. Traumatol. Surg. Res..

[6] Dellestable A., Cheval D., Kerfant N., Stindel E., Le Nen D., Letissier H. (2024). Long-Term Outcomes of Trapeziectomy with Gore-Tex^®^ Ligament Reconstruction for Trapezio-Metacarpal Osteoarthritis. Orthop. Traumatol. Surg. Res..

[7] Komura S., Hirakawa A., Masuda T., Nohara M., Kimura A., Matsushita Y., Matsumoto K., Akiyama H. (2022). Preoperative Prognostic Factors Associated with Poor Early Recovery after Trapeziectomy with Ligament Reconstruction and Tendon Interposition Arthroplasty for Thumb Carpometacarpal Osteoarthritis. Orthop. Traumatol. Surg. Res..

[8] Huang K., Hollevoet N., Giddins G. (2015). Thumb Carpometacarpal Joint Total Arthroplasty: A Systematic Review. J. Hand Surg. Eur. Vol..

[9] Ladd A.L., Weiss A.-P.C., Crisco J.J., Hagert E., Wolf J.M., Glickel S.Z., Yao J. (2013). The Thumb Carpometacarpal Joint: Anatomy, Hormones, and Biomechanics. Instr. Course Lect..

[10] Weiss A.-P.C., Goodman A.D. (2018). Thumb Basal Joint Arthritis. JAAOS-J. Am. Acad. Orthop. Surg..

[11] Ong F.R., Lim C.T., Goh J.C.H. (2009). Thumb Motion and Typing Forces during Text Messaging on a Mobile Phone. Proceedings of the 13th International Conference on Biomedical Engineering.

[12] Li Z.-M., Tang J. (2007). Coordination of Thumb Joints during Opposition. J. Biomech..

[13] Ellis B., Bruton A. (2002). A Study to Compare the Reliability of Composite Finger Flexion with Goniometry for Measurement of Range of Motion in the Hand. Clin. Rehabil..

[14] Gajdosik R.L., Bohannon R.W. (1987). Clinical Measurement of Range of Motion: Review of Goniometry Emphasizing Reliability and Validity. Phys. Ther..

[15] Zhao J.Z., Blazar P.E., Mora A.N., Earp B.E. (2020). Range of Motion Measurements of the Fingers via Smartphone Photography. Hand.

[16] Trejo Ramirez M.P., Evans N., Venus M., Hardwicke J., Chappell M. (2023). Reliability, Accuracy, and Minimal Detectable Difference of a Mixed Concept Marker Set for Finger Kinematic Evaluation. Heliyon.

[17] Reissner L., Fischer G., List R., Taylor W.R., Giovanoli P., Calcagni M. (2019). Minimal Detectable Difference of the Finger and Wrist Range of Motion: Comparison of Goniometry and 3D Motion Analysis. J. Orthop. Surg. Res..

[18] Luker K.R., Aguinaldo A., Kenney D., Cahill-Rowley K., Ladd A.L. (2014). Functional Task Kinematics of the Thumb Carpometacarpal Joint. Clin. Orthop. Relat. Res..

[19] Fischer G., Jermann D., List R., Reissner L., Calcagni M. (2020). Development and Application of a Motion Analysis Protocol for the Kinematic Evaluation of Basic and Functional Hand and Finger Movements Using Motion Capture in a Clinical Setting—A Repeatability Study. Appl. Sci..

[20] Sancho-Bru J.L., Jarque-Bou N.J., Vergara M., Pérez-González A. (2014). Validity of a Simple Videogrammetric Method to Measure the Movement of All Hand Segments for Clinical Purposes. Proc. Inst. Mech. Eng. Part H.

[21] American Society of Hand Therapists (2015). Clinical Assessment Recommendations.

[22] Gionfrida L., Rusli W.M.R., Bharath A.A., Kedgley A.E. (2022). Validation of Two-Dimensional Video-Based Inference of Finger Kinematics with Pose Estimation. PLoS ONE.

[23] Gu F., Fan J., Wang Z., Liu X., Yang J., Zhu Q. (2023). Automatic Range of Motion Measurement via Smartphone Images for Telemedicine Examination of the Hand. Sci. Prog..

[24] Kuchtaruk A., Yu S.S.Y., Iansavichene A., Davidson J., Wilson C.A., Symonette C. (2023). Telerehabilitation Technology Used for Remote Wrist/Finger Range of Motion Evaluation: A Scoping Review. Plast. Reconstr. Surg.–Glob. Open.

[25] Shinohara I., Inui A., Mifune Y., Yamaura K., Kuroda R. (2024). Posture Estimation Model Combined with Machine Learning Estimates the Radial Abduction Angle of the Thumb with High Accuracy. Cureus.

